# An Autobiographical Discussion

**Published:** 1918-10-12

**Authors:** 


### THE DOCTORS' POLITICAL MEETING.

### An Autobiographical Discussion.

The programme was certainly an attractive one. A highly respectable and much respected baronet in the chair, a medical Minister of Reconstruction in the leading oratorical *rôle*, and the object—to get members of the craft into Parliament. No wonder that the doctors turned up in their hundreds! Whether they went down to their houses justified or not is another question. They indeed learned something of interest to their profession. On authority they were told that the Ministry of Health Bill awaits only the final approval of the War Cabinet, that the Ministry is to be provided with a consultative medical council, and that the Bill does not propose a State Medical Service. Yet all this did not seem particularly relevant to the declared purpose of the meeting. Nor did the advice of the Minister of Reconstruction that success in public work demands preparation, patience, and ability to wait, appear much more helpful. What was desired was to get doctors into Parliament, and to "do it quick." Even the virtues which the Minister recited might exist without the accomplishment of the desired end. The counsel was excellent, but there still failed the men and the means and the method. The chairman, indeed, had a plan, and all the subsequent speakers were too polite to refer to it. Even its author rather regarded it as a "might have been." Had such a thing been possible, he would fain make the medical profession into an independent constituency, and the doctors could then directly elect the men of their choice. As nearly all the doctors already have votes as citizens, and many of them have a second vote as university graduates, the addition of a third vote might cause trouble to the politician who urged its adoption. Anyhow, the Minister of Reconstruction left it severely alone, and it did not reappear in the discussion. The chairman repeated it in a letter to the *Times* on the day after the meeting, but again there was no answering voice. The editor, it is true, discussed the main subject, and was so gracious as to allow that medical members of Parliament in recent times "have been accepted as desirable colleagues and as rather enhancing than lowering the prestige of the House," and this is doubtless more than he would say of certain other Parliamentary groups. But he provided no help in getting the matter forward. The enterprise had the editorial benediction, and its backers were advised to "choose the right men and to get them elected," which is exactly what the meeting was summoned to do. In short, just as the Minister of Reconstruction said "Be good," the editor of the *Times* says "Be clever"; and possibly there are members of the profession who regard each of these exhortations as superfluous. As for the chairman's patent scheme, none does it reverence, and this seems somewhat unkind.

With nothing more practical on hand the meeting considered the inevitable proposal of the appointment of a committee, and the organisers of the meeting had a scheme cut and dried. Their committee was to "nominate" suitable medical men and to further the election of those so nominated. But here the audience began to take a hand, and objections and suggestions and amendments began to multiply. The speaking was vigorous, and, if at times somewhat autobiographical, had a corresponding measure of personal interest. Thus one gentleman told us in pathetic tones that in his earlier years he had been banished to Western Africa because his proposals for improvements in the Medical Service of the Army had been in advance of his day; he himself was cultivating a constituency in the Black Country. Another speaker had formerly served in a Parliament existing, as he poetically described it, "on the fringes of the Empire," while a third had during the war given lectures to something like half a million of soldiers, and from a fourth we learned that the organisation of which he is the only true begetter was the real author of the Ministry of Health. These piquant details occupied some little time, and were, perhaps, hardly relevant to the debate. Indeed, it may be that they helped to produce confusion, for by this time the meeting was largely out of hand, and was hardly a model of Parliamentary decorum. But one thing was clear—namely, that the meeting would not have a committee endowed with authority to "nominate" suitable medical men as candidates for Parliament. Some voices called for a committee instructed to support medical candidates who might be "nominated," presumably in the usual legal fashion, while others favoured the mere preparation of a "scheme" to further the election of medical men to Parliament. Between these rival amendments, complicated with questions, withdrawals, rulings, and interruptions, to disentangle the issue was not easy, and even the reporters do not seem to be agreed on what was carried and what was rejected. But, whatever the committee is going to do, it has to report to a further meeting of the profession. Hence we may all be of good cheer, seeing that, according to one of the speakers, it is necessary to "do something." Having settled what the committee was to do, the next thing was to elect the members. Here again the organisers had their plan, and, indeed, they had printed it. Representatives from various professional organisations and eighteen "non-official members," these latter to be "elected by the meeting," though kindly nominated in advance by some "hidden hand"—thus was to be formed the "committee." The eighteen names were proposed and seconded by two of their number, and thus we had the authoritative voices of these gentlemen that the selection had been dictated by the highest motives.

Some little demur to so swift a bolus was not unnatural, and it found expression. But the real

**The Doctors' Political Meeting**—(*continued*).

explosion came when it was decreed that all persons who negected to vote should be counted as favouring the selected eighteen. This, indeed, was too much, and the chairman, gallantly struggling, was forced to the wall. Everyone had praise for his impartiality and confidence in his good faith, but his doctrine on the interpretation of absent ballot-papers had too much novelty to hope for immediate success. And so the great meeting ended, having, after long labours, appointed a committee! Thus travellers from Southampton and other remote areas may feel that they have not voyaged in vain. In the meantime rumours of an early general election harden, and it is always possible to be a day behind the fair.

